# Smartphone Overuse and Visual Impairment in Children and Young Adults: Systematic Review and Meta-Analysis

**DOI:** 10.2196/21923

**Published:** 2020-12-08

**Authors:** Jian Wang, Mei Li, Daqiao Zhu, Yang Cao

**Affiliations:** 1 School of Nursing Shanghai Jiao Tong University Shanghai China; 2 School of Medical Sciences Örebro University Örebro Sweden; 3 Clinical Epidemiology and Biostatistics School of Medical Sciences Örebro University Örebro Sweden

**Keywords:** visual impairment, smartphone, mobile phone, overuse, child, young adult, systematic review, meta-analysis

## Abstract

**Background:**

Smartphone overuse has been cited as a potentially modifiable risk factor that can result in visual impairment. However, reported associations between smartphone overuse and visual impairment have been inconsistent.

**Objective:**

The aim of this systematic review was to determine the association between smartphone overuse and visual impairment, including myopia, blurred vision, and poor vision, in children and young adults.

**Methods:**

We conducted a systematic search in the Cochrane Library, PubMed, EMBASE, Web of Science Core Collection, and ScienceDirect databases since the beginning of the databases up to June 2020. Fourteen eligible studies (10 cross-sectional studies and 4 controlled trials) were identified, which included a total of 27,110 subjects with a mean age ranging from 9.5 to 26.0 years. We used a random-effects model for meta-analysis of the 10 cross-sectional studies (26,962 subjects) and a fixed-effects model for meta-analysis of the 4 controlled trials (148 subjects) to combine odds ratios (ORs) and effect sizes (ES). The *I*^2^ statistic was used to assess heterogeneity.

**Results:**

A pooled OR of 1.05 (95% CI 0.98-1.13, *P*=.16) was obtained from the cross-sectional studies, suggesting that smartphone overuse is not significantly associated with myopia, poor vision, or blurred vision; however, these visual impairments together were more apparent in children (OR 1.06, 95% CI 0.99-1.14, *P*=.09) than in young adults (OR 0.91, 95% CI 0.57-1.46*,* *P*=.71). For the 4 controlled trials, the smartphone overuse groups showed worse visual function scores compared with the reduced-use groups. The pooled ES was 0.76 (95% CI 0.53-0.99), which was statistically significant (*P*<.001).

**Conclusions:**

Longer smartphone use may increase the likelihood of ocular symptoms, including myopia, asthenopia, and ocular surface disease, especially in children. Thus, regulating use time and restricting the prolonged use of smartphones may prevent ocular and visual symptoms. Further research on the patterns of use, with longer follow up on the longitudinal associations, will help to inform detailed guidelines and recommendations for smartphone use in children and young adults.

## Introduction

The use of smartphones has been increasing rapidly since their introduction in the late 2000s [1]. In 2019, the global smartphone penetration had reached approximately 41.5% of the global population [2]. Notably, the number of smartphone users in China was around 700 million in 2018, accounting for half of the Chinese population [3]. In addition, more than 80% of people in the United Kingdom owned or had ready access to a smartphone in 2019, representing a significant increase from 50% in 2012 [4]. Furthermore, more than 90% of young people between 16 and 34 years old in the United Kingdom owned a smartphone in 2019 [4].

With the continuous rise in youth digital media consumption, the incidence of ocular problems has also dramatically increased. A large portion of the population currently suffers from visual impairment, especially in Asian countries, with a rapidly increasing prevalence and younger age of onset [5-8]. It has been estimated that 49.8% (4.8 billion) and 9.8% (0.9 billion) of the global population will have myopia or high myopia by 2050 [9]. A recent study indicated that about 60 years ago, only 10%-20% of the Chinese population was nearsighted, but the percentage reached up to 90% of teenagers and young adults in 2015 [10]. Consistently, a school-based retrospective longitudinal cohort study (N=37,424 participants) found that the prevalence of myopia significantly increased from 56% in 2005 to 65% in 2015 [8].

Therefore, smartphone overuse among children and young adults has become a matter of crucial concern [11-13]. Several studies found increased use of digital devices in children aged 2-11 years old [14,15]. For example, a study including children aged 9-11 years from 12 countries showed that 54.2% of the children exceeded proposed screen time guidelines (≤2 hours per day) [15]. Compared with older people, children and young adults have greater risks of the undesirable consequences of smartphone overuse because they have less self-control in smartphone use [11]. A cross-sectional study (N=2639 participants) indicated that 22.8% of teenagers were addicted to smartphone use, which was related to hypertension [16]. Another study showed that users of mobile devices spent >20 hours weekly on email, text messages, and social networking services, indicating the heavy reliance on smartphones in their communication with other people [17]. Overall, smartphone overuse may result in significant harmful physical, psychological, and social consequences [18,19].

Some experimental studies have indicated that long-term use of a smartphone plays a key role in visual impairment, increasing the likelihood of poor vision [20-22]. For instance, a prospective clinical study (N=50 participants) showed that smartphone use for 4 hours resulted in a higher ocular surface disease index than that measured at baseline [20]. Kim et al [23] found that the increase of ocular symptoms extended to the general population, especially in adolescents, after expansion of smartphone use. However, other studies have reported the lack of evidence for such an association [24]. For example, a cross-sectional study (N=1153 participants) using stratified random cluster samples did not find a statistically significant association between smartphone use time and myopia [25]. Similarly, a study conducted in Ireland (N=418 participants) indicated that smartphone use time was not a risk factor for myopia [26]. Toh et al [27] found that smartphone use time was associated with an increased risk of visual symptoms (ie, blurring of vision, dry eye), but a decreased odds of myopia.

Despite increased concern about impaired vision due to smartphone overuse, existing quantitative evidence about the relationships between excessive smartphone use and visual impairment remains equivocal. Therefore, it is essential to confirm and quantify whether excessive smartphone use may result in visual impairment, especially in children and young adults.

The aim of this study was to conduct a systematic review and meta-analysis to summarize the existing evidence on the associations between smartphone overuse and visual impairment in children and young adults, which may further guide potential interventions to reduce the harmful impact of smartphone overuse on vision in this susceptible subpopulation.

## Methods

### Data Sources and Search Strategy

This systematic review and meta-analysis was based on a protocol designed in line with the standard Preferred Reporting Items for Systematic Reviews and Meta-Analysis (PRISMA) [28] and Meta-analysis of Observational Studies in Epidemiology (MOOSE) [29] criteria.

A systematic search was carried out in PubMed (US National Library of Medicine), Embase (Wolters Kluwer Ovid), Web of Science Core Collection (Clarivate Analytics), ScienceDirect (Elsevier), and Cochrane library (John Wiley & Sons, Ltd) for observational and experimental studies that investigated smartphone overuse or addiction in children (aged<18 years) or young people (aged<40 years), and its associations with impaired visual function such as myopia, poor vision, or blurred vision. To minimize publication bias, we also searched for additional studies in grey literature sources, including Virtual Health Library [30], NARCIS [31], Grey literature report [32], and Open grey EU [33]. The search was limited to publications published in English.

Free text and Medical Subject Headings (MeSH) terms were used for the search, including phone, smartphone, mobile/cell/cellular phone, electronic device, use, use time, screen time, overuse, addiction, eye, visual acuity, vision, vision screening, eyesight, myopia, myopic refraction, shortsighted/nearsighted/short sight, near sight, refraction errors, ocular/health effect, optic, blind, ophthalmology, optometry, retina, ametropia/amblyopia symptom, visual assessment, and visual problem (see Multimedia Appendix 1 for the complete search strategy). We included all observational studies and controlled trials (randomized or nonrandomized) addressing smartphone use and visual impairment in humans since the beginning of the databases up to June 2020. Furthermore, manual retrieval was performed following the initial database search to ensure the inclusion of the latest literature.

### Inclusion and Exclusion Criteria

All observational and experimental studies were included if they fulfilled the following criteria: (1) original studies examining the use of a smartphone (or mobile phone) and eyesight, including population-based longitudinal studies, cohort studies, case-control studies, cross-sectional studies, and controlled clinical trials; (2) participants are children aged ≤18 years or young people aged ≤40 years (a young adult was defined as the developmental stage between 18 and 40 years [34,35]); (3) reported frequency or time of smartphone use (in minutes or hours, or per day or week); (4) the endpoint of interest is the incidence of visual impairment or decline, including myopia, poor vision, blurred vision, various visual function scores indicating impaired vision, or other unspecific visual impairments; and (5) vision measurements of the groups are provided to calculate the effect size (ES) of visual impairment or odds ratio (OR) for the risk of visual impairment, as well as the associated 95% CIs or other data to estimate the variance or accuracy (eg, standard error).

Studies were excluded if they: (1) were narrative reviews, editorial papers, commentaries, letters, or methodological papers; (2) evaluated visual function with no reliable/relevant estimates for smartphone use; (3) no reference or control group was included in the analysis; and (4) animal studies.

### Data Extraction

After the systematic search of the relevant articles in the databases, two investigators (JW and ML) embarked on screening and identification of potentially relevant abstracts independently. For any disagreements that occurred between the two investigators regarding the eligibility of a study, there was a thorough discussion or advice from an academic expert (YC). Subsequently, articles for selected abstracts were downloaded, and data were extracted by JW and YC independently using a standardized form in Microsoft Excel. The extracted data were compared and summarized to obtain one final document from which the analysis was conducted. The information extracted included: name of first author, year of publication, study design, duration of study, country that the study was conducted in, eyesight measurement, smartphone use time, smartphone use frequency, sample size, incidence of cases with impaired vision, outcome ascertainment method, OR or ES and the associated 95% CI, and statistical analysis method used.

### Study Quality Assessment

The Joanna Briggs Institute (JBI) Critical Appraisal Checklist for Analytical Cross Sectional Studies, JBI Appraisal Checklist for Quasi-Experimental Studies, and JBI Critical Appraisal Checklist for Randomized Controlled Trials were used to assess the quality of the studies included in the meta-analysis [36]. JW and YC assessed the quality of the articles independently and the final assessment was achieved upon discussion (Multimedia Appendix 2).

### Statistical Analysis

For studies that did not report the OR, it was calculated using the numbers of cases with and without visual impairment of the reference/control group and overuse group. For studies that measured visual impairment using continuous variables, ES was calculated as the difference between the means divided by the pooled SD as follows [37]:

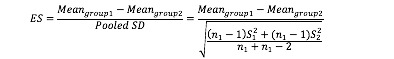



where *n*_1_ and *n*_2_, and *S*_1_ and *S*_2_ are the sample sizes and standard deviations for group 1 and group 2, respectively.

A positive ES indicates a worse visual function. Heterogeneity of the included studies was investigated using the *I*^2^ statistic [38], in which *I*^2^>30% was considered to indicate moderate heterogeneity and *I*^2^>50% was considered to indicate substantial heterogeneity [39]. A *P* value <.05 from the noncentral chi-squared test for heterogeneity was considered to indicate statistically significant heterogeneity [40]. The contribution of each study to the heterogeneity and their influence on the pooled OR or ES were assessed using the Baujat plot [41]. The pooled ORs with corresponding 95% CIs were calculated using random-effects models owing to heterogeneity among the studies and are presented using forest plots [42]. The possibility of publication bias was assessed by the combination of the Egger test and visual inspection of the funnel plot [43].

Subgroup analysis was performed for the cross-sectional studies according to the outcome of visual impairment (myopia, poor vision, or blurred vision) and mean age of the subjects (children, ≤18 years; young people, 18-40 years). Leave-one-out (LOO) analysis was also performed to investigate the influence of a single study on the pooled effect as an additional sensitivity analysis [44].

A two-sided *P* value <.05 of the pooled estimates was considered statistically significant unless otherwise specified. All analyses were performed in R 4.0.0 (R Foundation for Statistical Computing, Vienna, Austria) using the packages meta 4.12-0 [45] and dmetar 0.0.9000 [46].

## Results

### Characteristics of Included Studies

**Figure 1:**
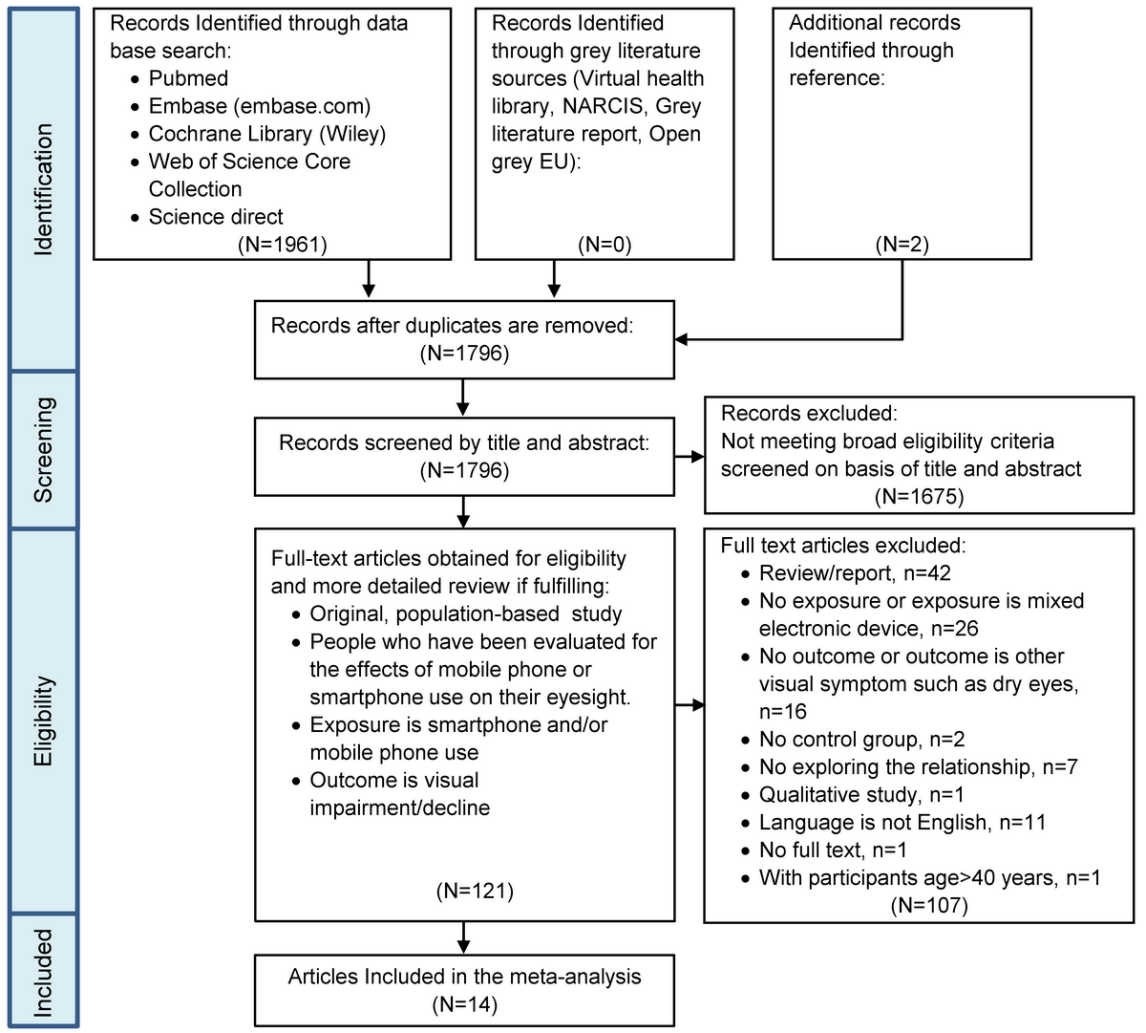
PRISMA (Preferred Reporting Items for Systematic Reviews and Meta-Analyses) flow diagram for screening and selection of articles on smartphone overuse and visual impairment in children and young adults.

In total, 1961 articles were obtained from all of the databases. After removing duplicates, 1796 articles remained, 121 of which were considered to be relevant for the meta-analysis after screening of titles and abstracts. After screening the full text of the 121 articles downloaded, 14 articles met our inclusion criteria, including 10 cross-sectional studies and 4 controlled trials, comprising a total of 27,110 participants with mean ages ranging from 9.5 to 26.0 years. The flowchart of article searching and screening is shown in Figure 1. The 10 cross-sectional studies addressed incidents of myopia [24-27,47], blurred vision [48-50], and poor vision and other unspecified visual impairments [23,27,48,51]. In our analysis, the unspecified visual impairments were treated as poor vision. There were 2 studies [27,48] that addressed two visual impairment outcomes, and each outcome was treated as a single study in the meta-analysis. The 4 studies that used a controlled trial design assessed the ocular surface disease index score [20], asthenopia score [21], oculomotor function [52], and viewing distance [22]. A more detailed summary of the characteristics of the included studies and the main outcomes is provided in Table 1 and Table 2, respectively.

**Table 1:** General characteristics of the included studies.

Reference	Year	Country	Study design	Age of participants (years), mean (SD) or range	Sampling method	N participants
Küçer et al [49]	2008	Turkey	Cross-sectional	University students (age not given)	Convenience sample	229
Toh et al [27]	2019	Singapore	Cross-sectional	13.3 (2.0)	Matrix-stratified sample	1884
Merrie et al [51]	2019	Ethiopia	Cross-sectional	13.1 (2.8)	Multistage sampling	601
Guan et al [47]	2019	China	Cross-sectional	10.6 (1.15)	Randomly selected sample	19,934
Kim et al [23]	2016	Korea	Cross-sectional	15 (0.9)	Convenience sample	715
Liu et al [24]	2019	China	Cross-sectional	9.5 (2.1)	Stratified cluster sample	566
Meo et al [50]	2005	Saudi Arabia	Cross-sectional	26.0 (13.4)	Voluntary (response) sample	873
Alharbi et al [48]	2019	Saudi Arabia	Cross-sectional	21.8 (2.4)	Random sample	605
Huang et al [25]	2019	China	Cross-sectional	19.6 (0.9)	Stratified random cluster sample	1153
McCrann et al [26]	2020	Ireland	Cross-sectional	16.8 (4.4)	Voluntary sample	402
Antona et al [21]	2018	Spain	RCT^a^	23.7 (2.6)	Random sample	54
Choi et al [20]	2018	South Korea	CT^b^	26.0 (3.0)	Nonrandomized sample	50
Lee et al [52]	2019	Korea	CT	20-29	Voluntary sample	26
Long et al [22]	2017	Australia	CT	21.5 (3.3)	Voluntary sample	18

^a^RCT: randomized controlled trial.

^b^CT: controlled trial.

**Table 2:** Outcomes and results of the included studies.

Reference	Response rate	Exposure; type of measure	Outcome; type of measure	Main results
Küçer et al [49]	100%	Time of mobile phone possession; Q^a^	Blurred vision; Q	≤2 years: 8.8% (4/45) >2 years: 27.2% (50/184)
Toh et al [27]	93.78% (1884/2009)	Time of smartphone use (per hour); Q	(1) Myopia; Q (2) Poor vision/visual impairment; Q	(1) OR^b^ 0.97 (95% CI 0.94-0.99) (2) OR 1.05 (95% CI 1.02-1.08)
Merrie et al [51]	95.09% (601/632)	Duration of mobile exposure; Q	Poor vision/visual impairment; objective assessment	>2 h/day: 6.6% (18/271) ≤ 2 h/day: 7.5% (20/265)
Guan et al [47]	UK^c^	Time of smartphone use; Q	Visual acuity; objective assessment	1 h/day: 20% (117/584); ≤1 h/day: 18% (3492/19350)
Kim et al [23]	97.41% (715/734)	Time of smartphone use; Q	Poor vision/ocular symptom score; Q	>2 h/day: 72% (260/360); ≤2 h/day: 52% (170/327)
Liu et al [24]	88.7% (566/638)	Time of smartphone use (per hour); Q	Myopia; objective assessment	OR 0.90 (95% CI 0.57-1.43)
Meo et al [50]	100%	Use of mobile phone (duration of calls); Q	Blurred vision; Q	>0.5 h/day: 5% (5/100); ≤0.5 h/day: 5.23% (39/746)
Alharbi et al [48]	93.1% (605/650)	Duration of Smartphone use per day; Q	(1) Poor vision; Q (2) Blurred vision; Q	(1) >3 h/day: 57.2% (270/472); ≤3 h/day: 45.9% (61/133） (2) >3 h/day: 46.0% (217/472); ≤3 h/day: 57.1% (76/133）
Huang et al [25]	96.08% (1153/1200)	Duration of daily smartphone use; Q	Myopia; objective assessment	>3 h/day: 84.57% (296/350); ≤ 3 h/day: 88.03% (537/610)
McCrann et al [26]	96.17% (402/418)	Time on phone (minutes/day); Q	Myopia; Q	OR 1.026 (95% CI 1.001-1.051)
Antona et al [21]	100%	Smartphone reading vs printed hardcopy reading	Asthenopia score; Q	27.96 (SD 20.11) vs 13.25 (SD 12.76)
Choi et al [20]	100%	Smartphone use after 4 hours vs baseline	Ocular surface disease index scores; Q	25.03 (SD 10.61) vs 15.08 (SD 8.83)
Lee et al [52]	86.67% (26/30)	Smartphone use 20 minutes vs 5 minutes	Oculomotor function; Q	6.35 (SD 3.54) vs 3.73 (SD 4.09)
Long et al [22]	100%	Using smartphone after 1 hour vs baseline	Viewing distance; objective assessment	27.8 (SD 7.7) cm vs 31 (SD 8.2) cm

^a^Q: questionnaire.

^b^OR: odds ratio.

^c^UK: unknown.

### Association Between Smartphone Overuse and Incidence of Visual Impairment

**Figure 2:**
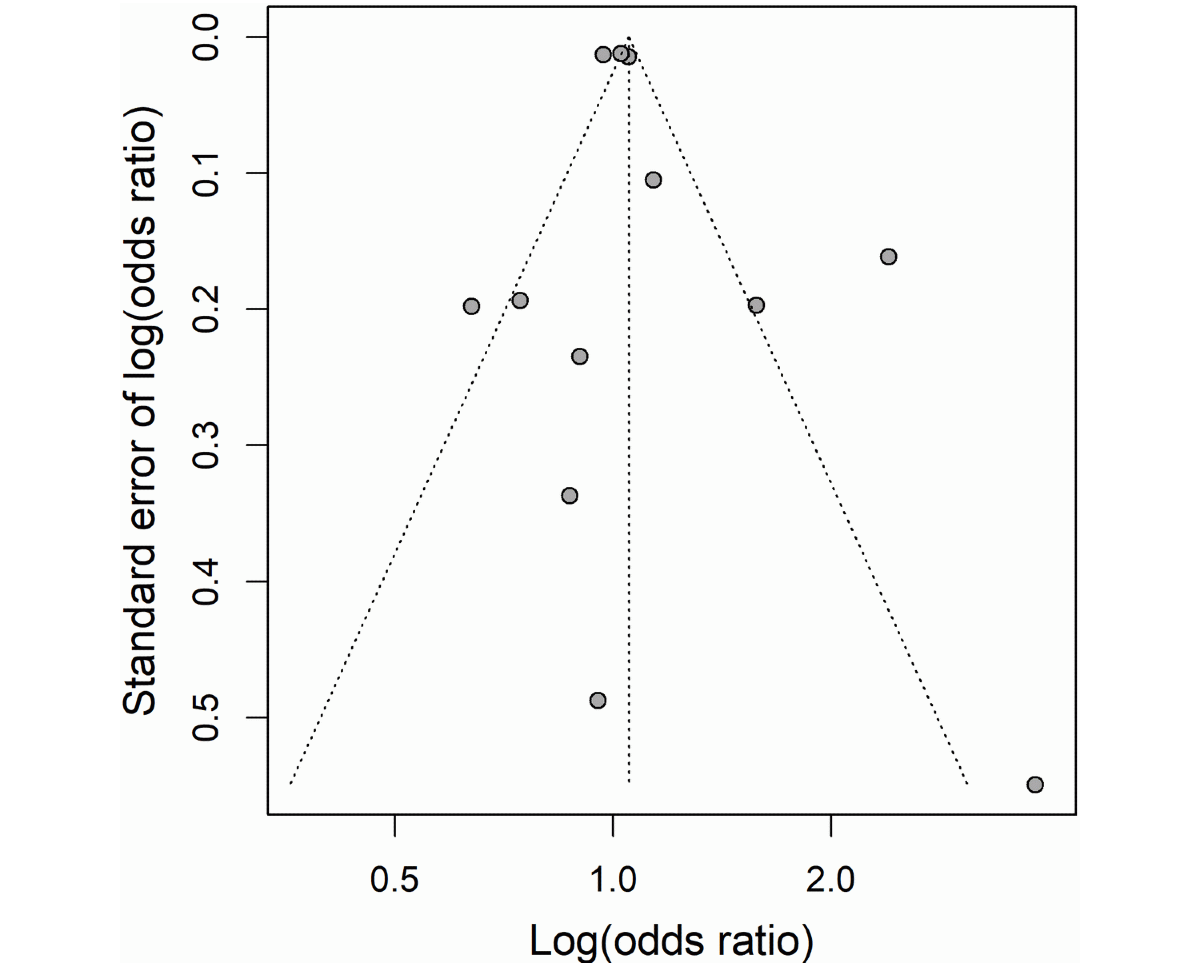
Funnel plot with pseudo 95% confidence limit for cross-sectional studies.

The funnel plot of ORs for the included cross-sectional studies appeared to be symmetric (Figure 2). Although ORs from two studies [23,49] showed slight bias with other studies, no statistically significant publication bias was found based on the Egger test (*P*=.43).

**Figure 3:**
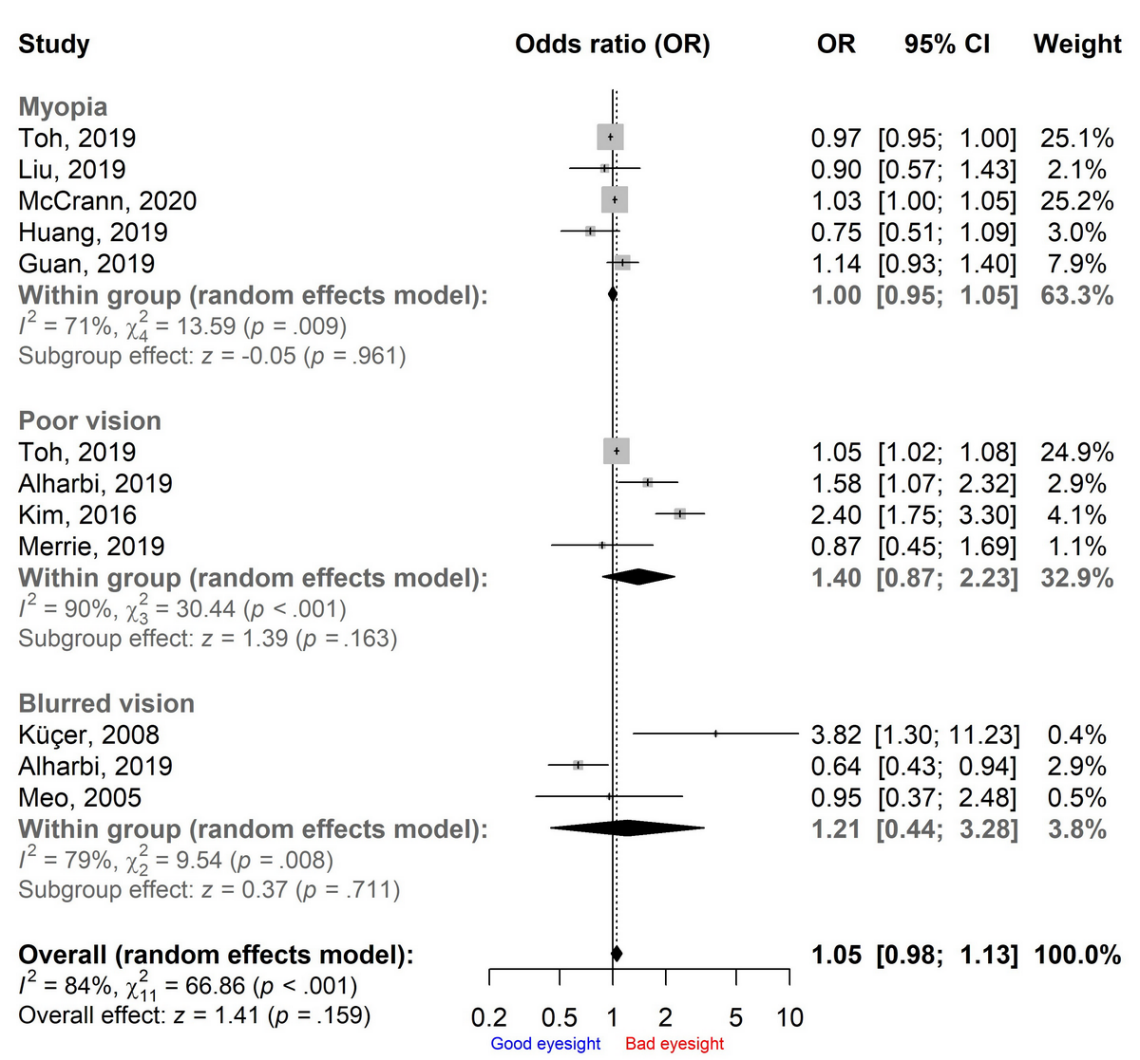
Pooled odds ratios (ORs) of visual impairment in the smartphone overuse group compared to the reduced-use group.

**Figure 4:**
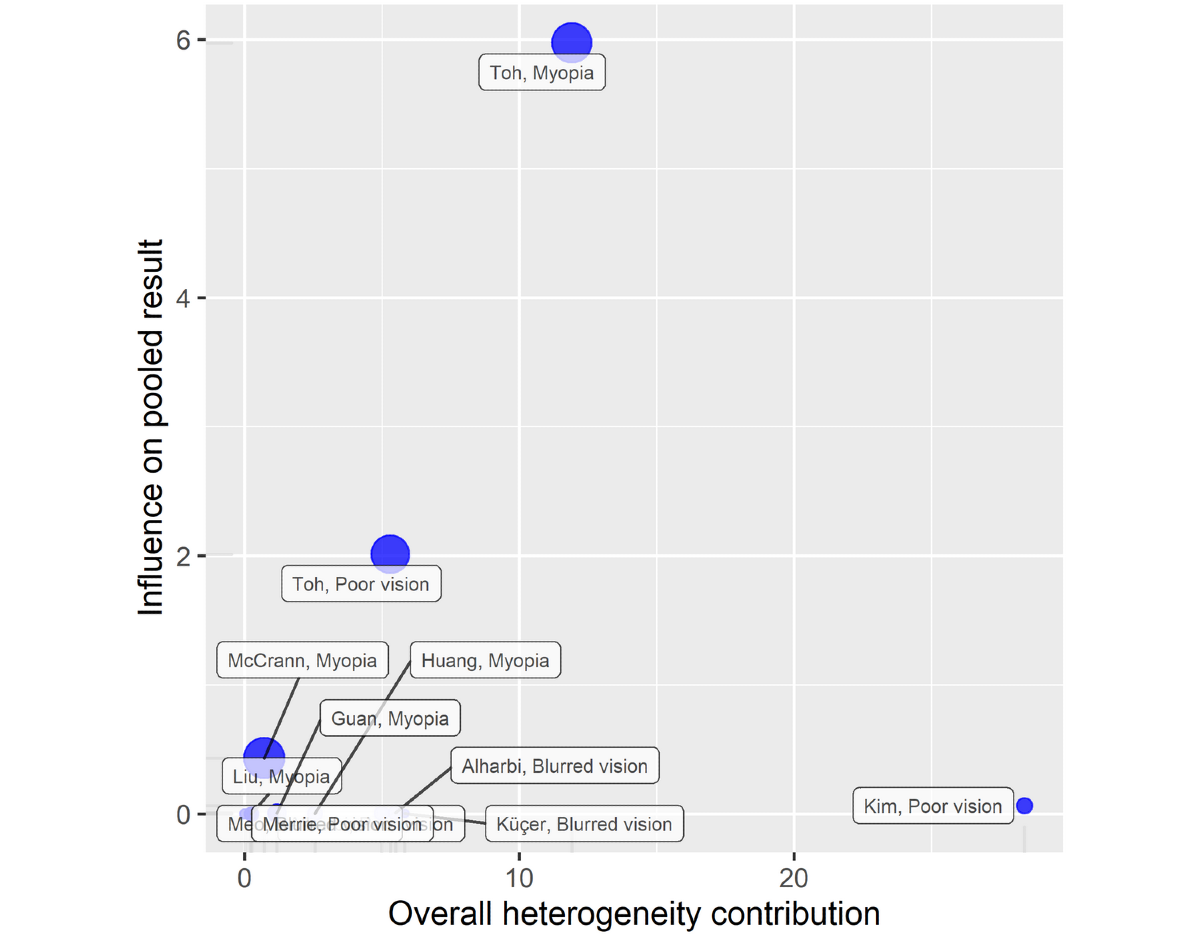
Baujat plot for cross-sectional studies.

Statistically significant heterogeneity was present among the ORs on visual impairment incidence (*I*^2^=84%, *P*<.001; Figure 3). The Baujat plot indicated that the study of Kim et al [23] substantially contributed to the heterogeneity but had a minimal influence on the pooled OR (Figure 4). Overall, although the pooled OR showed that the odds of visual impairment was higher for the smartphone overuse group compared to the reduced-use group (OR 1.05, 95% CI 0.98-1.13), the result was not statistically significant (*P*=.16; Figure 3). None of the pooled ORs for specific visual impairment was significant in subgroup analyses. The pooled ORs for myopia, poor vision, and blurred vision were 1.00 (95% CI 0.95-1.05), 1.40 (95% CI 0.87-2.23), and 1.21 (95% CI 0.44-3.28), respectively (Figure 3). The pooled OR was not statistically significant in either of the age subgroups, which was 1.06 (95% CI 0.99-1.14, *P*=.09) for children and 0.91 (95% CI 0.57-1.46, *P*=.71) for young adults.

**Figure 5:**
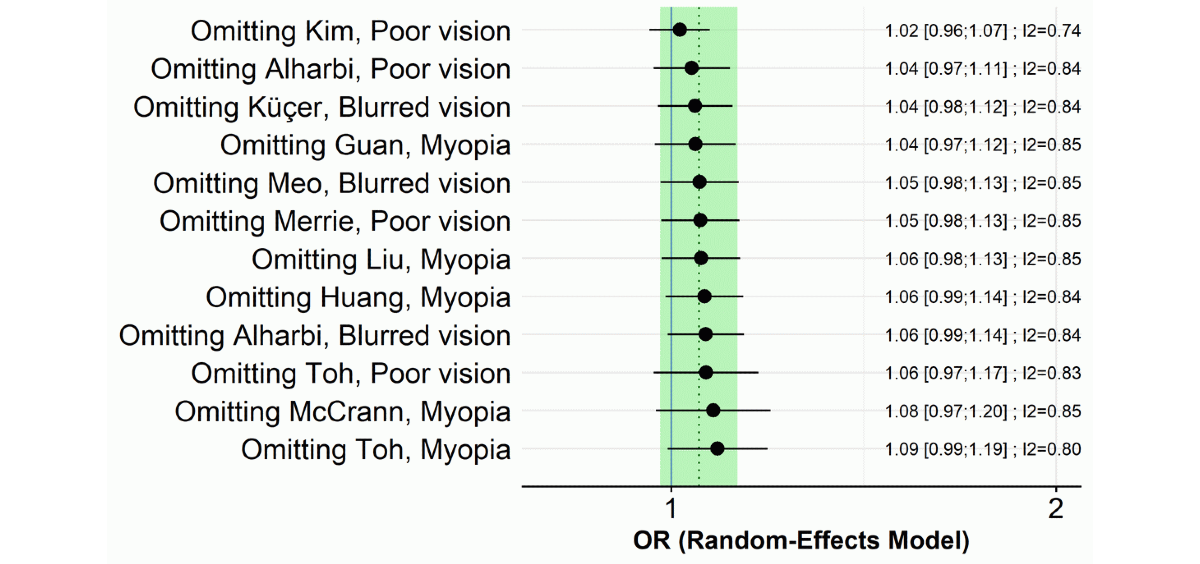
Pooled odds ratios (ORs) of visual impairment in the smartphone overuse group compared to the reduced-use group from leave-one-out analysis.

The LOO sensitivity test indicated that ORs of visual impairment in the smartphone overuse group compared to the reduced-use group ranged from 1.02 to 1.09; however, none of the ORs was statistically significant (Figure 5).

### Smartphone Overuse Associated With Worse Visual Function Scores

**Figure 6:**
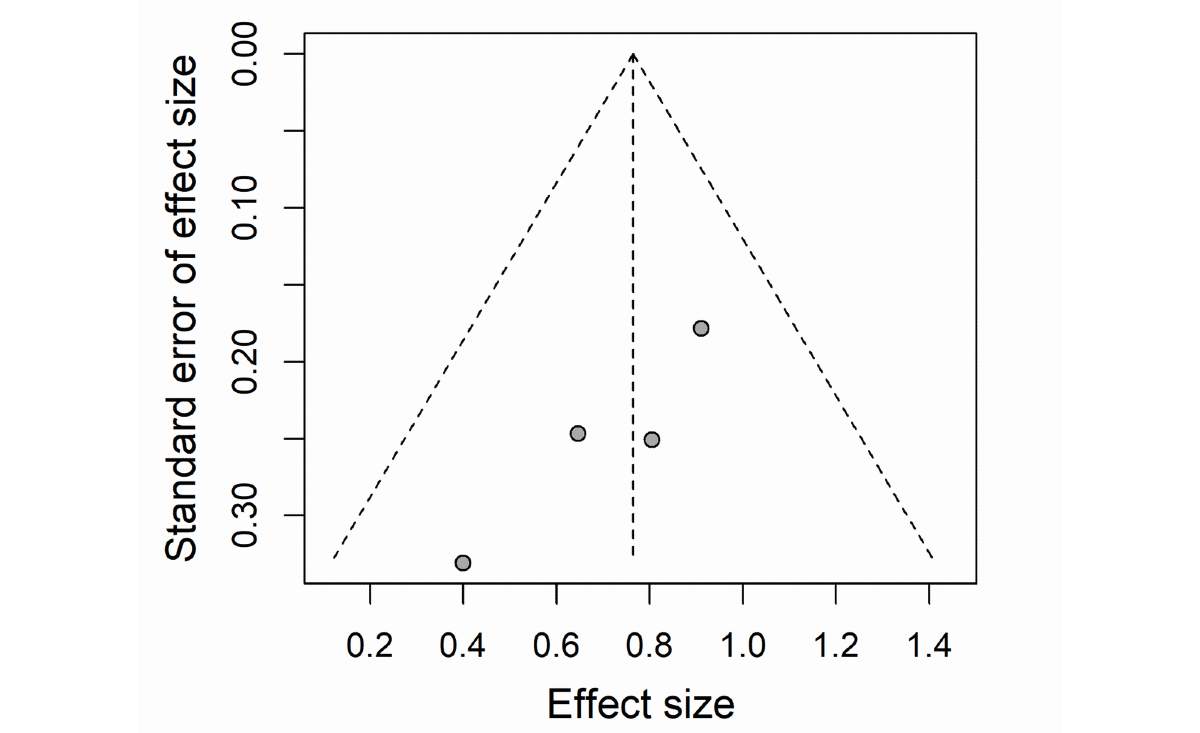
Funnel plot with pseudo 95% confidence limit for controlled trials.

**Figure 7:**
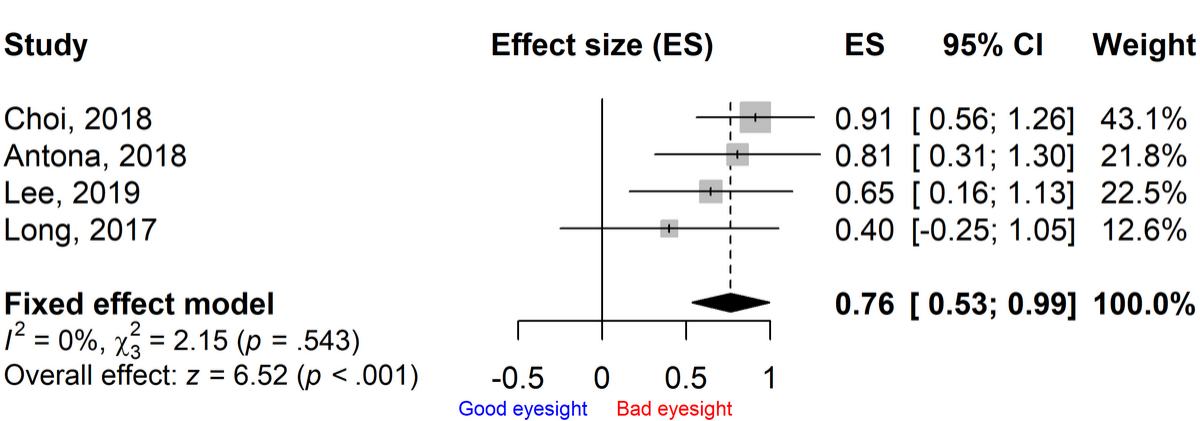
Pooled effect size (ES) of visual function score in the smartphone overuse group compared to the reduced-use group.

The funnel plot of ES for the included controlled trials appeared to be symmetric (Figure 6), and no statistically significant publication bias was found by the Egger test (*P*=.067). No statistically significant heterogeneity was present among the ESs on visual impairment incidence (*I*^2^=0%, *P*=.54; Figure 7).

In all of the controlled trials, the smartphone overuse group showed worse visual function scores than the reduced-use group, with ESs ranging from 0.40 to 0.91 (Figure 7). The pooled ES was 0.76 (95% CI 0.53-0.99), which was statistically significant (*P*<.001), indicating that compared with the reduced-use group, the visual function score in the smartphone overuse group was 0.76 SD worse (Figure 7).

**Figure 8:**
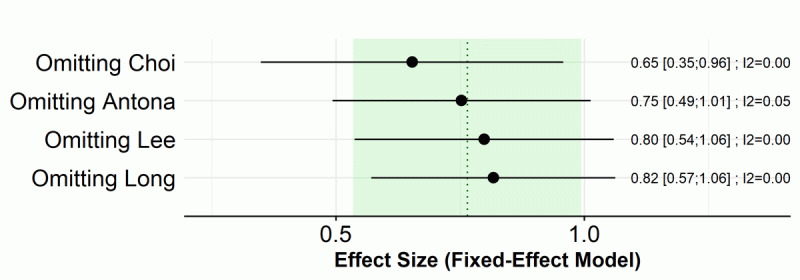
Pooled effect sizes (ESs) of visual function score in the smartphone overuse group compared to the reduced-use group from leave-one-out analysis.

The LOO sensitivity test indicated that the results are robust, with the ESs ranging from 0.65 to 0.82, and all of the ESs were statistically significant (Figure 8).

## Discussion

### Principal Findings

The purpose of this systematic review and meta-analysis was to summarize currently available evidence with reference to the relationship between smartphone overuse and visual impairment in children and young adults. Among the 14 studies included in the analysis, 9 found a significant association between smartphone overuse and visual impairment. Our pooled results showed negative but not statistically significant associations (OR=1.05, 95% CI 0.98-1.13) between smartphone overuse and myopia, blurred vision, or poor vision in the included cross-sectional studies. However, the adverse effect was more apparent in children (OR=1.06, 95% CI 0.99-1.14) than in young adults (OR=0.91, 95% CI 0.57-1.46). We also found that smartphone overuse may cause worse visual function than reduced use in the included controlled trials (ES=0.76, 95% CI 0.53-0.99). As the results are mixed, further studies are warranted. To our knowledge, this is the first systematic review that comprehensively summarized existing data on smartphone overuse and visual impairment in children and young adults.

There are several possible reasons for the lack of a statistically significant association observed between smartphone overuse and visual impairment when pooling cross-sectional studies. First, most of the existing studies included in this systematic review were from Asia, which has higher prevalence rates of visual impairment. The myopia prevalence in East Asia was already reported to be high before the introduction of digital devices [53]. Previous studies indicated that myopia prevalence increased more rapidly in people with more years of education and intensive schooling without particular exposure to screen devices [54-56]. For example, a study conducted in Singapore found that myopia prevalence increased more rapidly among individuals who started elementary school after the 1980s [57]. Consistently, a study in Israel found that teenage boys who attended Orthodox schools had much higher rates of myopia than students from other schools who spent less time reading books in the 1990s [53]. Therefore, education and intensive schooling may have a large contribution to the increase in myopia prevalence [58]. Recent studies have also extensively described the relationship between education and visual impairment [59]. Furthermore, a high prevalence of myopia from Taiwan was found in cohorts with low exposure to digital devices [56]. Thus, it is still debatable whether smartphone overuse would lead to a higher risk of myopia or other visual problems.

Second, most of the studies included in this analysis divided smartphone overuse as use time over 2 or 3 hours per day. However, there is some evidence that the time people actually spend engaged with a digital screen is far longer [60-62], suggesting that people may use other electronic devices. Overuse of other digital devices might also play an important role in visual impairment. Some studies have explored the relationships between digital screen time (eg, computer, tablet, smartphone, or other handheld electronic screens) and visual impairment [58,61,63-66]. For instance, a birth cohort study (N=5074 participants) showed that increased computer use was associated with myopia development in children [65]. Yang et al [63] found that screen exposure was significantly and positively associated with preschool myopia, which is consistent with the results of another cohort study [66]. However, the results of studies assessing the impacts of screen time on visual impairment have been mixed. A recent systematic review showed that screen time was not significantly associated with the prevalence and incidence of myopia [58], which may largely support our pooled result of cross-sectional studies. Thus, the relationship needs to be further validated. Moreover, given differences in the use of various digital devices, some studies have compared the impacts of smartphone use with other digital devices on visual impairment [20,24,25,27]. These results are also inconsistent. For instance, Guan et al [47] (N=19,934 participants) found that prolonged (>60 minutes/day) computer usage and smartphone usage were both significantly associated with greater refractive error. Nevertheless, Liu et al [24] and Huang et al [25] found that myopia in children was not associated with time spent using various electronic devices, including smartphones, tablets, and computers. By contrast, a study with a representative sample of 1884 adolescents showed that smartphone use time was associated with an increased risk of visual symptoms, but no significant association was found for tablet use [27]. A controlled trial (N=50 participants) indicated that the smartphone use group had higher fatigue, burning, and dryness scores than the computer use group [20]. Although the existing research supports that smartphone use might cause worse vision than other digital devices, further convincing evidence is needed to support this conclusion owing to the low number of studies.

Third, several studies have shown that technology use or screen time alone is of minimal risk to visual impairment, whereas more time spent outdoors is related to a reduced risk of myopia and myopic progression [25,67,68]. However, there is also evidence that the increased use of digital devices is associated with more time at work and less time spent outdoors, resulting in a substitution effect [58,69]. For example, Dirani et al [69] reported that the lack of adequate outdoor activity might be related to the rise in digital screen time. More specifically, recent educational screen time might be a replacement for reading or writing, in addition to recreational screen time (eg, computer or video games) [69]. For instance, smartphones are used by children mainly for playing games (29%) and watching videos (20%) but also for learning (19%) [70]. Thus, digital screen time might not be a causal factor, but may be a substitute for a different types of work [58]. There is also some evidence that children 9-11 years old who spent less than 2 hours playing on a computer were 1.98 times more likely to spend more than 1 hour outside than those reporting 2 or more hours of computer use [71]. Although these results might reflect a tradeoff between outdoor time and digital screen time, with screen time being a proxy for indoor time, there is no evidence to confirm this substitution effect [58]. Thus, further studies in this field are warranted.

Besides the findings in the cross-sectional studies, we also found that the smartphone overuse group presented worse visual function scores than the reduced-use group in each of the included controlled trials and in the pooled result. Biologically, the effects of smartphones on ocular symptoms can be explained by two types of electromagnetic fields (EMFs): extremely low-frequency EMFs and radiofrequency (RF) electromagnetic radiation (EMR) [72,73]. The intensity of radiation from mobile phones is relatively low with a specific absorption rate <4 W/kg [72,74]. However, it has been reported that adverse effects such as DNA damage and thickening of the cornea occur even at a specific absorption rate lower than 4 W/kg [72,75,76]. The local specific absorption rate has been shown to be higher in tissues at a younger age, suggesting higher susceptibility of adolescents to smartphones [77]. The EMFs generated by smartphones may interact with the tissues of the eyes [73,78], which may cause apoptosis, cataract formation, edema, endothelial cell loss, inflammatory responses, and neurological effects [72,74,79,80]. The RF EMR may affect the body thermally and nonthermally [81], which may result in oxidative stress in the cornea and the lens [74]. These effects by EMFs and RF EMR on the eyes, especially on the cornea and the lens, could suggest why ocular symptoms such as blurring, redness, visual disturbance, inflammation, and lacrimation increase with more exposure to smartphones [23]. Although experimental studies may provide causal inferences, our result needs to be further confirmed due to the limited number of existing studies.

Regarding the association between smartphone overuse and myopia examined in the cross-sectional studies, multiple ocular symptoms found in the experimental studies do not necessarily reflect pathological changes in the eyes, such as myopia. Few longitudinal cohort studies have examined the impacts of screen exposure on myopia, and the results are inconsistent [66,82]. To our knowledge, there have been no experimental or longitudinal studies detecting the impacts of smartphone overuse on myopia specifically. Thus, a longitudinal cohort study design establishing the temporal sequence of prior exposure to environmental factors would be useful to examine whether smartphone overuse may increase the risk of developing myopia.

In addition, the heterogeneity was high in the meta-analysis of included cross-sectional studies. First, a large number of studies have identified potential risk factors that may result in visual impairment, which included both genetic and environmental factors [20,26,56,58] such as age [26], education and occupation [58], outdoor activity [20,58], and parental myopia [20]. However, some studies did not include these variables in the multivariate analysis, which might contribute to the inconsistent findings, and might further affect the individual effect estimates and the pooled OR. Second, some studies only used univariate analysis to investigate the associations between smartphone use time and visual impairment [47,69], which might hinder the exploration of their interrelationships. Third, the assessment of the outcome was inconsistent. For example, some studies used a self-reported questionnaire to identify myopia [26,27], while others used an objective assessment [24,25]. Furthermore, the division of smartphone overuse was inconsistent, which may have precluded us from determining their significant relationships. A guideline advised limiting recreational screen time to no more than 2 hours per day [83]. Therefore, further studies in this field should use a broadly recognized standard to define smartphone overuse.

There are also other limitations of this study that need to be addressed. All of the included studies used a self-reported questionnaire to evaluate smartphone use time. Participants in the included experimental studies also mostly reported their visual function using questionnaires. The questionnaires themselves may be a potential source of error due to inaccurate reporting or recall bias of the participants. Further research should adopt objective instruments to measure smartphone use time and visual acuity screening to examine visual function. Furthermore, generalization of the results should be interpreted with caution owing to the low number of studies included in each meta-analysis. Limiting the review to studies reported in English may have also resulted in nonreporting of studies published in other languages. Nevertheless, our review involved rigorous methodological procedures to obtain and pool data from 27,110 children and young adults. We also adopted a wide range of search terms to retrieve all potential articles published in English, including the grey literature, which might have helped to reduce the publication bias in the final combination.

### Conclusions

Overall, current evidence suggests that the results of the association between smartphone overuse and visual impairment in children and young adults are mixed. Although the statistically significantly negative association between smartphone overuse and visual impairment in the meta-analysis was only confirmed in controlled trials and not in cross-sectional studies, the adverse effect of smartphone overuse on visual functions was more apparent in children. However, these relationships need to be further verified. Further research on the patterns of use, with longer follow-up periods to detect longitudinal associations, and the exact mechanisms underlying these associations will help inform detailed guidelines for smartphone use in children and young adults. In addition, understanding the factors of smartphone overuse that account for the risk of ocular symptoms could help the growing population of smartphone users, especially children and young adults, to use smartphones in a healthier manner.

## Appendix

Multimedia Appendix 1 Literature search strategy and results.

Multimedia Appendix 2 Tables of study quality assessment.

## Abbreviations

EMF: electromagnetic field
EMR: electromagnetic radiation
ES: effect size
JBI: Joanna Briggs Institute
LOO: leave-one-out
OR: odds ratio
RF: radiofrequency
