# Supplementary material for: Smartphone Overuse and Visual Impairment in Children and Young Adults: Systematic Review and Meta-Analysis
Source: J Med Internet Res. 2020 Dec 8;22(12):e21923. doi: 10.2196/21923 (PMC7755532; doi:10.2196/21923)
Supplement: Multimedia Appendix 1 [file jmir_v22i12e21923_app1.docx]

Literature search strategy and results

1. Pubmed

| Interface: pubmed.com  Number of hits: 613 | Field labels   - exp/ = exploded MeSH term - / = non exploded MeSH term - .ti,ab. = title, abstract and author keywords - adjx = within x words, regardless of order - * = truncation of word for alternate endings |
| --- | --- |
| (((((((((((((((eyesight[Title/Abstract]) OR (myopia[Title/Abstract])) OR (‘visual acuity’[Title/Abstract])) OR (vision[Title/Abstract])) OR (‘myopic refraction’[Title/Abstract])) OR (shortsighted[Title/Abstract])) OR (nearsighted[Title/Abstract])) OR (‘short sight’[Title/Abstract])) OR (‘near sight’[Title/Abstract])) OR (‘refraction errors’[Title/Abstract])) OR (‘visual impairment’[Title/Abstract])) OR (‘eye dysfunction’[Title/Abstract])) OR (‘eye disease’[Title/Abstract])) OR (‘eye disorder’[Title/Abstract])) OR (‘eye function’[Title/Abstract]))  AND  ((((((((phone*[Title/Abstract]) OR (smartphone*/[Title/Abstract])) OR (‘mobile phone’[Title/Abstract])) OR (‘cell phone’[Title/Abstract])) OR (‘cellular phone’[Title/Abstract])) OR (‘screen time’[Title/Abstract])) OR (‘electronic device’[Title/Abstract])) OR (‘digital device’[Title/Abstract]))  ((((( "Eye Diseases"[Mesh]) OR "Refractive Errors"[Mesh]) OR "Vision, Ocular"[Mesh]) OR "Vision Screening"[Mesh]) OR "Myopia"[Mesh])  AND  (((("Cell Phone"[Mesh]) OR "Cell Phone Use"[Mesh]) OR "Smartphone"[Mesh]) OR "Screen Time"[Mesh])  After removing duplicates: n=613. | |

2. Embase

| Interface: embase.com  Number of hits: 1076  Comment: Emtree is the controlled vocabulary in Embase | Field labels   - /exp = exploded Emtree term - /de = non exploded Emtree term - ti,ab = title and abstract - NEAR/x = within x words, regardless of order - * = truncation of word for alternate endings |
| --- | --- |
| #1:phone*:ti,ab,kw OR smartphone:ti,ab,kw OR 'mobile phone':ti,ab,kw OR 'screen time':ti,ab,kw OR 'electronic device':ti,ab,kw OR 'digital device':ti,ab,kw OR 'cell phone use':ti,ab,kw  #2:eyesight:ti,ab,kw OR myopia:ti,ab,kw OR 'visual acuity':ti,ab,kw OR vision:ti,ab,kw OR 'myopic refraction':ti,ab,kw OR shortsighted:ti,ab,kw OR nearsighted:ti,ab,kw OR 'short sight':ti,ab,kw OR 'near sight':ti,ab,kw OR 'refraction errors':ti,ab,kw OR 'visual impairment':ti,ab,kw OR 'eye dysfunction':ti,ab,kw OR 'eye disease':ti,ab,kw OR 'eye function':ti,ab,kw  #3: #1 AND #2.  #1: ('smartphone'/exp OR 'smartphone') AND [humans]/lim  #2: ('mobile phone'/exp OR 'mobile phone') AND [humans]/lim  #3: ('cell phone use'/exp OR 'cell phone use') AND [humans]/lim  #4: ('screen time'/exp OR 'screen time') AND [humans]/lim  #5: ('vision'/exp OR 'vision') AND [humans]/lim  #6: ('myopia'/exp OR 'myopia') AND [humans]/lim  #7: ('visual acuity'/exp OR 'visual acuity') AND [humans]/lim  #8: ('visual impairment'/exp OR 'visual impairment') AND [humans]/lim  #9: ('refraction error'/exp OR 'refraction error') AND [humans]/lim  #10: #1 AND #2 AND #3 AND #4  #11: #5 AND #6 AND #7 AND #8 AND #9  #12: #10 AND #11  After removing duplicates: n=1076. | |

3. Cochrane Library

| Interface: cochranelibrary.com  Number of hits: 67 | Field labels   - ti,ab,kw = title, abstract and author keywords - exp/ = exploded MeSH term - NEAR/x = within x words, regardless of order - * = truncation of word for alternate endings |
| --- | --- |
| #1 MeSH descriptor: [Smartphone] explode all trees  #2 MeSH descriptor: [Cell Phone] explode all trees  #3 MeSH descriptor: [Screen Time] explode all trees  #4 MeSH descriptor: [Eye Diseases] explode all trees  #5 MeSH descriptor: [Vision, Ocular] explode all trees  #6 MeSH descriptor: [Myopia] explode all trees  #7 MeSH descriptor: [Visual Acuity] explode all trees  #8 MeSH descriptor: [Refractive Errors] explode all trees  #9 ('electronic device'):ti,ab,kw OR ('digital device'):ti,ab,kw OR ('mobile phone'):ti,ab,kw  #10 (eyesight):ti,ab,kw OR ('eye dysfunction'):ti,ab,kw OR ('eye function'):ti,ab,kw OR (visual impairment):ti,ab,kw AND ('short sight'):ti,ab,kw  #11 #1 or #2 or #3 or #9  #12 #4 or #5 or #6 or #7 or #8 or #10  #13 #11 and #12 | |

4. Web of Science Core Collection

| Interface: webofknowledge.com  Number of hits: 179 | Field labels   - Title = title - NEAR/x = within x words, regardless of order - * = truncation of word for alternate endings |
| --- | --- |
| #1: TITLE: (eyesight) OR TITLE: (myopia) OR TITLE: ('eye function') OR TITLE: ('visual acuity') OR TITLE: (vision) OR TITLE: ('eye disorder') OR TITLE: ('myopic refraction') OR TITLE: (shortsighted) OR TITLE: (nearsighted) OR TITLE: ('short sight') OR TITLE: ('near sight') OR TITLE: ('refraction errors') OR TITLE: ('eye disease') OR TITLE: ('eye function') OR TITLE: ('eye dysfunction') OR TITLE: ('visual impairment')    #2: TITLE: (phone*) OR TITLE: (smartphone*) OR TITLE: ('mobile phone') OR TITLE: ('cell phone') OR TITLE: ('cellular phone') OR TITLE: ('screen time') OR TITLE: ('electronic device') OR TITLE: ('digital device')  #3: #1 AND #2 | |

5. Science Direct

| Interface: sciencedirect.com  Date of Search: 7 May 2020  Number of hits: 26 | Field labels   - ti,ab = title and abstract - NEAR/x = within x words, regardless of order - * = truncation of word for alternate endings |
| --- | --- |
| #1: (phone* OR smartphone* OR 'mobile phone' OR 'cell phone' OR 'cellular phone' OR 'screen time' OR 'digital device')  #2: (eyesight OR myopia OR 'visual acuity' OR vision OR 'myopic refraction' OR 'short sight' OR eye OR 'refraction error')  #3: #1 AND #2 | |
