# Supplementary material for: Smartphone Overuse and Visual Impairment in Children and Young Adults: Systematic Review and Meta-Analysis
Source: J Med Internet Res. 2020 Dec 8;22(12):e21923. doi: 10.2196/21923 (PMC7755532; doi:10.2196/21923)
Supplement: Multimedia Appendix 2 [file jmir_v22i12e21923_app2.doc]

Supplemental Table S1. Study quality analysis for included articles in systematic review exploring the impacts of smartphone on eyesight (JBI Critical Appraisal Checklist for Analytical Cross Sectional Studies, 2017)

| Validity questions | Toh^[1]^ | Liu^[2]^ | Alharbi^[3]^ | Küçer^[4]^ | Meo^[5]^ | Huang^6]^ | Kim^[7]^ | Guan^[8]^ | Merrie^[9]^ | McCrann^[10]^ |
| --- | --- | --- | --- | --- | --- | --- | --- | --- | --- | --- |
| 1. Were the criteria for inclusion in the sample clearly defined? | Y | Y | Y | Y | Y | Y | Y | Y | Y | Y |
| 2. Were the study subjects and the setting described in detail? | Y | Y | Y | Y | Y | Y | Y | Y | Y | Y |
| 3. Was the exposure measured in a valid and reliable way? | Y | Y | N | Y | Y | N | Y | Y | UN | Y |
| 4. Were objective, standard criteria used for measurement of the condition? | N | Y | N | N | N | Y | N | Y | Y | N |
| 5. Were confounding factors identified? | Y | Y | Y | N | N | Y | Y | Y | Y | Y |
| 6. Were strategies to deal with confounding factors stated? | Y | Y | N | N | N | Y | Y | Y | Y | Y |
| 7. Were the outcomes measured in a valid and reliable way? | N | Y | N | N | N | Y | Y | Y | Y | N |
| 8. Was appropriate statistical analysis used? | Y | Y | Y | Y | Y | Y | Y | Y | Y | Y |
| Overall quality rating | H | H | M | M | M | H | H | H | H | H |
| Overall appraisal | Include | Include | Include | Include | Include | Include | Include | Include | Include | Include |

N, no; NA, not applicable; H, high study quality; M, medium study quality; UN, unclear; Y, yes.

Supplemental Table S2. Study quality analysis for included articles in systematic review exploring the impacts of smartphone on eyesight (JBI Critical Appraisal Checklist for Quasi-Experimental Studies (non-randomized experimental studies), 2017)

| Validity questions | Choi^[11]^ | Lee^[12]^ | Long^[13]^ |
| --- | --- | --- | --- |
| 1. Is it clear in the study what is the‘cause’ and what is the ‘effect’ (i.e. there is no confusion about which variable comes first)? | Y | Y | Y |
| 2. Were the participants included in any comparisons similar? | Y | Y | Y |
| 3. Were the participants included in any comparisons receiving similar treatment/care, other than the exposure or intervention of interest? | Y | Y | Y |
| 4. Was there a control group? | Y | N | N |
| 5. Were there multiple measurements of the outcome both pre and post the intervention/exposure? | Y | Y | Y |
| 6. Was follow up complete and if not, were differences between groups in terms of their follow up adequately described and analyzed? | Y | Y | N |
| 7. Were the outcomes of participants included in any comparisons measured in the same way? | Y | Y | Y |
| 8. Were outcomes measured in a reliable way? | Y | Y | Y |
| 9. Was appropriate statistical analysis used? | Y | Y | Y |
| Overall quality rating | H | H | H |
| Overall appraisal | Include | Include | Include |

N, no; NA, not applicable; H, high study quality; M, medium study quality; UN, unclear; Y, yes.

Supplemental Table S3. Study quality analysis for included articles in systematic review exploring the impacts of smartphone on eyesight (JBI Critical Appraisal Checklist for Randomized Controlled Trials, 2017)

| Validity questions | Antona^[14]^ |
| --- | --- |
| 1. Was true randomization used for assignment of participants to treatment groups? | UN |
| 2. Was allocation to treatment groups concealed? | N |
| 3. Were treatment groups similar at the baseline? | Y |
| 4. Were participants blind to treatment assignment? | NA |
| 5. Were those delivering treatment blind to treatment assignment? | NA |
| 6. Were outcomes assessors blind to treatment assignment? | NA |
| 7. Were treatment groups treated identically other than the intervention of interest? | Y |
| 8. Was follow up complete and if not, were differences between groups in terms of their follow up adequately described and analyzed? | Y |
| 9. Were participants analyzed in the groups to which they were randomized? | Y |
| 10. Were outcomes measured in the same way for treatment groups? | Y |
| 11. Were outcomes measured in a reliable way? | Y |
| 12. Was appropriate statistical analysis used? | Y |
| 13. Was the trial design appropriate, and any deviations from the standard RCT design (individual randomization, parallel groups) accounted for in the conduct and analysis of the trial? | Y |
| Overall quality rating | H |
| Overall appraisal | Include |

N, no; NA, not applicable; H, high study quality; M, medium study quality; UN, unclear; Y, yes.

References:

[1] Toh S H, Coenen P, Howie E K, et al. Mobile touch screen device use and associations with musculoskeletal symptoms and visual health in a nationally representative sample of Singaporean adolescents[J]. Ergonomics, 2019, 62(6): 778-793.

[2] Liu S, Ye S, Xi W, et al. Electronic devices and myopic refraction among children aged 6-14 years in urban areas of Tianjin, China[J]. Ophthalmic & physiological optics : the journal of the British College of Ophthalmic Opticians (Optometrists), 2019, 39(4): 282-293.

[3] Alharbi M T, Alem A A, Alrizgi H A, et al. IMPACT OF SMARTPHONES ON EYE HEALTH AMONG HEALTH SCIENCES STUDENTS IN TAIBAH UNIVERSITY, SAUDI ARABIA[J]. Indo American Journal of Pharmaceutical Sciences, 2019, 6(2): 3523-3530.

[4] Küçer N. Some ocular symptoms experienced by users of mobile phones[J]. Electromagnetic biology and medicine, 2008, 27(2): 205-209.

[5] Meo S A, Al-Drees A M. Mobile phone related-hazards and subjective hearing and vision symptoms in theSaudi population[J]. International Journal of Occupational Medicine and Environmental Health, 2005, 18(1): 53-57.

[6] Huang L M, Kawasaki H, Liu Y Q, et al. The prevalence of myopia and the factors associated with it among university students in Nanjing: A cross-sectional study[J]. Medicine, 2019, 98(10): 7.

[7] Kim J, Hwang Y, Kang S, et al. Association between Exposure to Smartphones and Ocular Health in Adolescents[J]. Ophthalmic Epidemiology, 2016, 23(4): 269-276.

[8] Guan H, Yu N N, Wang H, et al. Impact of various types of near work and time spent outdoors at different times of day on visual acuity and refractive error among Chinese school-going children[J]. PloS one, 2019, 14(4): e0215827-e0215827.

[9] Merrie Y A, Tegegne M M, Munaw M B, et al. Prevalence And Associated Factors Of Visual Impairment Among School-Age Children In Bahir Dar City, Northwest Ethiopia[J]. Clinical optometry, 2019, 11: 135-143.

[10] Mccrann S. Smartphone use as a possible risk factor for myopia[J]. J Med Internet Res, 2020.

[11] Choi J H, Li Y, Kim S H, et al. The influences of smartphone use on the status of the tear film and ocular surface[J]. PLoS ONE, 2018, 13(10).

[12]Lee D, Hong S, Jung S, et al. The effects of viewing smart devices on static balance, oculomotor function, and dizziness in healthy adults[J]. Medical Science Monitor, 2019, 25: 8056-8060.

[13]Long J, Cheung R, Duong S, et al. Viewing distance and eyestrain symptoms with prolonged viewing of smartphones[J]. Clinical & experimental optometry, 2017, 100(2): 133-137. [14] Antona B, Barrio A R, Gascó A, et al. Symptoms associated with reading from a smartphone in conditions of light and dark[J]. Applied ergonomics, 2018, 68: 12-17.

[14]Antona, B., Barrio, A. R., Gascó, A., Pinar, A., González-Pérez, M., & Puell, M. C. (2018). Symptoms associated with reading from a smartphone in conditions of light and dark. Applied ergonomics, 68, 12-17. doi: 10.1016/j.apergo.2017.10.014
